# Stronger Sexual Selection in Warmer Waters: The Case of a Sex Role Reversed Pipefish

**DOI:** 10.1371/journal.pone.0044251

**Published:** 2012-08-27

**Authors:** Nuno M. Monteiro, David O. Lyons

**Affiliations:** 1 Centro de Investigação em Biodiversidade e Recursos Genéticos, Vairão, Portugal; 2 CEBIMED, Faculdade de Ciências da Saúde, Universidade Fernando Pessoa, Porto, Portugal; 3 National Parks and Wildlife Service, Galway, Ireland; University of California, Berkeley, United States of America

## Abstract

In order to answer broader questions about sexual selection, one needs to measure selection on a wide array of phenotypic traits, simultaneously through space and time. Nevertheless, studies that simultaneously address temporal and spatial variation in reproduction are scarce. Here, we aimed to investigate the reproductive dynamics of a cold-water pipefish simultaneously through time (encompassing variation within each breeding cycle and as individuals grow) and space (by contrasting populations experiencing distinct water temperature regimes) in order to test hypothesized differences in sexual selection. Even though the sampled populations inhabited locations with very different water temperature regimes, they exhibited considerable similarities in reproductive parameters. The most striking was the existence of a well-defined substructure in reproductive activity, where larger individuals reproduce for longer periods, which seemed dependent on a high temperature threshold for breeding rather than on the low temperatures that vary heavily according to latitude. Furthermore, the perceived disparities among populations, such as size at first reproduction, female reproductive investment, or degree of sexual size dimorphism, seemed dependent on the interplay between seawater temperature and the operational sex ratio (OSR). Contrary to our expectations of an enhanced opportunity for sexual selection in the north, we found the opposite: higher female reproductive investment coupled with increased sexual size dimorphism in warmer waters, implying that a prolonged breeding season does not necessarily translate into reduced sexual selection pressure. In fact, if the limited sex has the ability to reproduce either continuously or recurrently during the entire breeding season, an increased opportunity for sexual selection might arise from the need to compete for available partners under strongly biased OSRs across protracted breeding seasons. A more general discussion on the effects of climate change in the pressure of sexual selection is also presented.

## Introduction

During recent decades, sexual selection has been acknowledged as a major evolutionary force able to explain both micro- and macro-evolutionary patterns [1]. Despite active research in this field, a unified measure of sexual selection remains elusive [2]. The most widely used measures, such as the operational sex-ratio (OSR), the opportunity for sexual selection, or the Bateman gradient, even though useful (e.g., [1], [3]), all have been have been shown to be flawed for general application (e.g., [1]). In order to answer broader questions about sexual selection, one needs to evaluate selection on a wide array of phenotypic traits, a task that requires a thorough understanding of a species' reproductive dynamics through space and time. Indeed, few studies have examined reproductive variation over large geographic scales (e.g., [4], [5]) or within a reproductive season (e.g., [6], [7]). Studies that simultaneously address temporal and spatial variation in reproduction are even scarcer, probably due to the underlying logistics. Nevertheless, the rewards promised by such an approach – exploring reproduction along a “space-time” continuum – are potentially substantial, namely by helping to discriminate between patterns that are intimately related to or occurring independently of latitude. Also, in specific cases where latitude significantly covaries with temperature, such an approach will help us understand the influence of temperature variation on seasonally dynamic reproductive parameters, easing the prediction of the impact of climate change on sexual selection processes, a topic that remains largely ignored [8], [9]. Finally, an approach that simultaneously combines both spatial and temporal data will ultimately provide an opportunity to test the robustness of commonly used measures of selection, such as the OSR, in depicting the dynamics of sexual selection.

**Figure 1:**
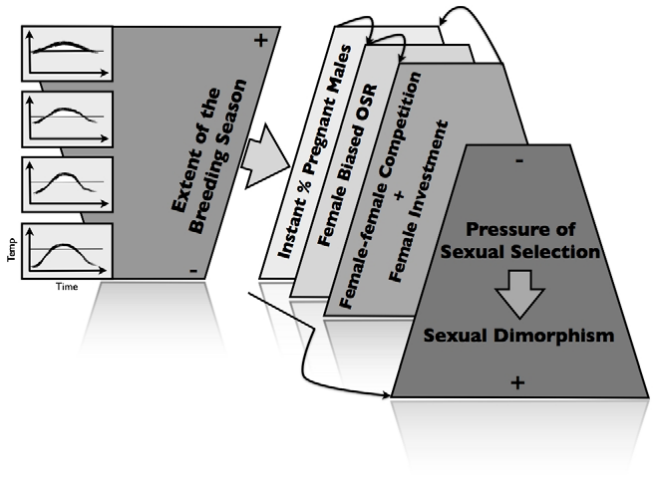
The hypothesized effect of increasing seawater temperatures on the worm pipefish breeding season length and potential consequences in terms of sexual selection pressure. Shorter breeding seasons should imply less available males, as most will become pregnant rapidly, causing a female biased OSR that, in turn, should influence the degree of female-female competition and investment in reproduction. Increased competition and investment should further reduce the number of still available males, as in a positive feedback loop. As a result, sexual selection pressure is expected to increase, a pattern that may be reflected in the degree of sexual dimorphism.

Here, we aimed to investigate reproductive dynamics through time (by portraying the variation occurring within each breeding cycle and as individuals grow) and space (by contrasting populations inhabiting different latitudes, with distinct water temperature regimes) in order to test hypothesized differences in the pressure of sexual selection. We have analyzed data from two natural populations of the worm pipefish, *Nerophis lumbriciformis* (Pisces, Syngnathidae), a species with sex-role reversal, sexual dimorphism and, as with all syngnathids, male pregnancy [10]. This data set provided a rare opportunity to identify common reproductive patterns in two populations experiencing very different seawater temperature regimes, thus allowing for the determination of differential expression patterns related to latitude (e.g. density of sexually mature individuals, extent of the breeding season, OSR, egg investment, pregnancy rates or sexual size dimorphism). Given that the extent of the breeding season as well as fecundity are expected to be reduced in cold waters [11], we hypothesize that the southern worm pipefish population, experiencing warmer waters and longer breeding seasons, will face a milder pressure of sexual selection, correlated with less female-biased OSRs. Additionally, we predict that a population living further north, where males will be on high demand, will show an increased female investment in mating competition (Figure 1). Since there is now increasing evidence that variation between individuals is both common and dynamically important for demography (e.g., [12]), we further test for cohort-specific differences in reproduction within each of the selected locations, and examine evidence of asymmetries between populations. Finally, given that our dataset gathers new information on the ecology of two geographically distant worm pipefish populations, this will be an invaluable opportunity to examine if this sex-role reversed system complies with some of the most well known rules of ecotypic variation that scrutinize the geographical variation of characters that are conceivably under sexual selection. Specifically, we will test for the relationship between sexual selection pressure and large-scale patterns on body size variation such as those described in Bergmann's rule (larger individuals in colder environments, [13]) and Rensch's rule (decrease in sexual size dimorphism with increasing size when females are the larger sex, [14]), as well as physiological processes related to reproduction, such as Rass's rule (increase in egg size with decreasing water temperatures, [15]).

## Materials and Methods

### Study species

**Figure 2:**
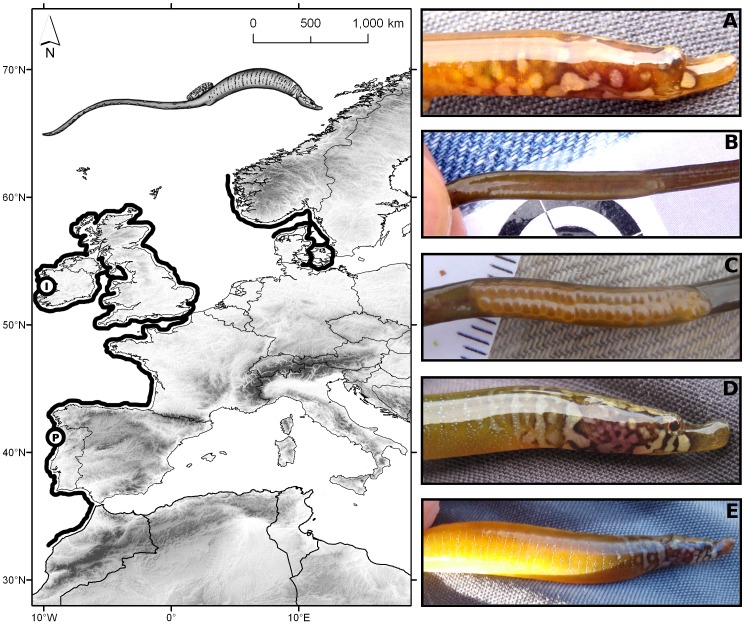
The biogeographical distribution of *Nerophis lumbriciformis*, according to Dawson (1986). Circles indicate the location of the two sampled populations (P =  Portugal, Viana do Castelo, 41°70′N 8°85′W; I =  Ireland, Galway Bay, 53°15' N 9°10′W). Pictures of adult worm pipefish are also presented (A- Male's facial pigmentation; B- Male's empty brooding surface; C- Male's brooding surface with eggs; D- Female's facial pigmentation; E- Female′s keel-like structure on the abdominal region).

*Nerophis lumbriciformis* is amongst the smallest of the Western European pipefish species (Figure 2). It can be usually found in coastal rocky areas, from the intertidal to approximately 30 metres [16], inhabiting complex substrata formed by seaweed covered boulders [17]. Its distribution ranges from the western coast of Norway to the Kattegat and from Belgium southwards to Morocco [16](Figure 2). The worm pipefish feeds on small vagile epiphytic crustaceans [18], sucking them through its distinctive upwards-pointing snout. This species is sexually dimorphic (Figure 2), with females actively courting males during the courtship and mating rituals [19], and also presenting more intense colorations in the head and trunk areas [20]. Moreover, as females grow, a keel-like structure starts to develop in the trunk region where a distinctive striped-pattern is amplified during inter- or intra-gender interactions. Males, with a more uniform coloration, present a conspicuous abdominal brooding area where eggs are incubated. Female egg posturing consists of depositing a band of tightly compacted bright orange sticky eggs to the male's incubating surface that are in direct contact with the surrounding seawater. A male receives eggs from one female only, but females can potentially mate with multiple males. Pelagic larvae emerge after approximately 30 days and no further parental care is provided [21].

### Sampling methodology

Worm pipefish were collected by hand from below the mean low-water mark, monthly during spring tides, on rocky shores in Ireland (Galway Bay: 53°15′N 9°10′W; 1998–1999) and Portugal (Viana do Castelo: 41°70′N 8°85′W; 1997–2009). Collected pipefish were measured to the nearest millimeter (L_T_). Additionally, for pregnant males, we collected information on egg number and size as well as the area of the brooding surface whenever possible. Also, in the Irish population, mature (ready to spawn) oocytes were counted within female ovaries. Detailed information on sampling methodologies and pipefish migration patterns to the intertidal can be found in [10], [22] and [18], [23].

### Statistical analysis

The sex ratio (proportion of males) was averaged for each month in each location. A similar procedure was used for the comparison of the OSR, the proportion of non-pregnant males among all individuals available to mate at any given time and place [24]. As such, we excluded already pregnant males, as suggested by Kvarnemo & Ahnesjö [25] when specifically addressing pipefish.

Size is a central variable in syngnathid reproduction [26], so we opted to summarize information by comparing either the largest or the smallest reproductively mature pipefish. Looking at clearly distinct size classes [27] is a good way of identifying changes in reproductive strategies, occurring as fish grow, that could otherwise be obscured by simultaneously presenting information for all size classes. Following the same methodology used in previous works [28]–[30], we have defined size cut-offs for ‘large’ and ‘small’ individuals as half of the standard deviation (SD) below and above the mean size for each sex, both in Ireland (♂: mean = 12.16 cm, *SD* = 1.19 cm; ♀: mean = 12.44cm; *SD* = 1.46 cm) and Portugal (♂: mean = 11.26 cm, *SD* = 1.25 cm; ♀: mean = 11.74 cm; *SD* = 1.41 cm).

For the estimation of a relationship between male size and i) the annual percentage of pregnancy or ii) the average brooding surface area, we used one-centimetre size classes (but pipefish larger than 15 cm were pooled within the > = 14 cm size class as they were rare).

To test for differences between the two sampled populations in selection pressure on males, standardized selection differentials [S_males_, the difference in average male size before selection (all males) and after selection (pregnant males) divided by the standard deviation of all male size [31]] were also calculated for the months when all male size classes were breeding in the two populations (February to July).

In order to detect size differences between the sexes and sampled locations, a two-way ANOVA was conducted [2 factors, each with two levels: sex (male or female) and sampling site (Ireland or Portugal)]. Even though mild heteroscedasticity was observed in our data, since there was no correlation between means and standard deviations and the visual inspection of residuals suggested no major disruptions, we opted to continue with the analysis given that the *F* statistic is quite robust against this violation [32]. Otherwise, non-parametric tests were used when appropriate, namely: a) Wilcoxon matched pairs tests to compare pipefish densities between Ireland and Portugal, monthly captures of males and females in each location, and differences in the estimated S_males_ between populations; b) Sign tests to analyse differences in the sex-ratios (SR and OSR); c) a Kruskall-Wallis ANOVA by ranks to analyse differences in oocytes between large, intermediate and small females; and d) Friedman ANOVAs to evaluate differences in the eggs carried by large, intermediate or small males, analysed separately for each population.

No specific permits were required for the described field studies as the locations were not privately owned or protected and the sampling did not involve endangered or protected species.

## Results

Overall, a total of 1767 adult worm pipefish were sampled in the intertidal area: 940 in Portugal (519♂ and 421♀) and 827 in Ireland (451♂ and 376♀). The total number of sampled individuals was similar in both locations (*χ^2^ = *0.082, *DF* = 3, *P* = 0.752) and no apparent difference in overall pipefish densities in the intertidal was observed (Wilcoxon matched-pairs test, using average monthly captures: *N* = 12, *Z* = 0.471, *P* = 0.638).

**Figure 3:**
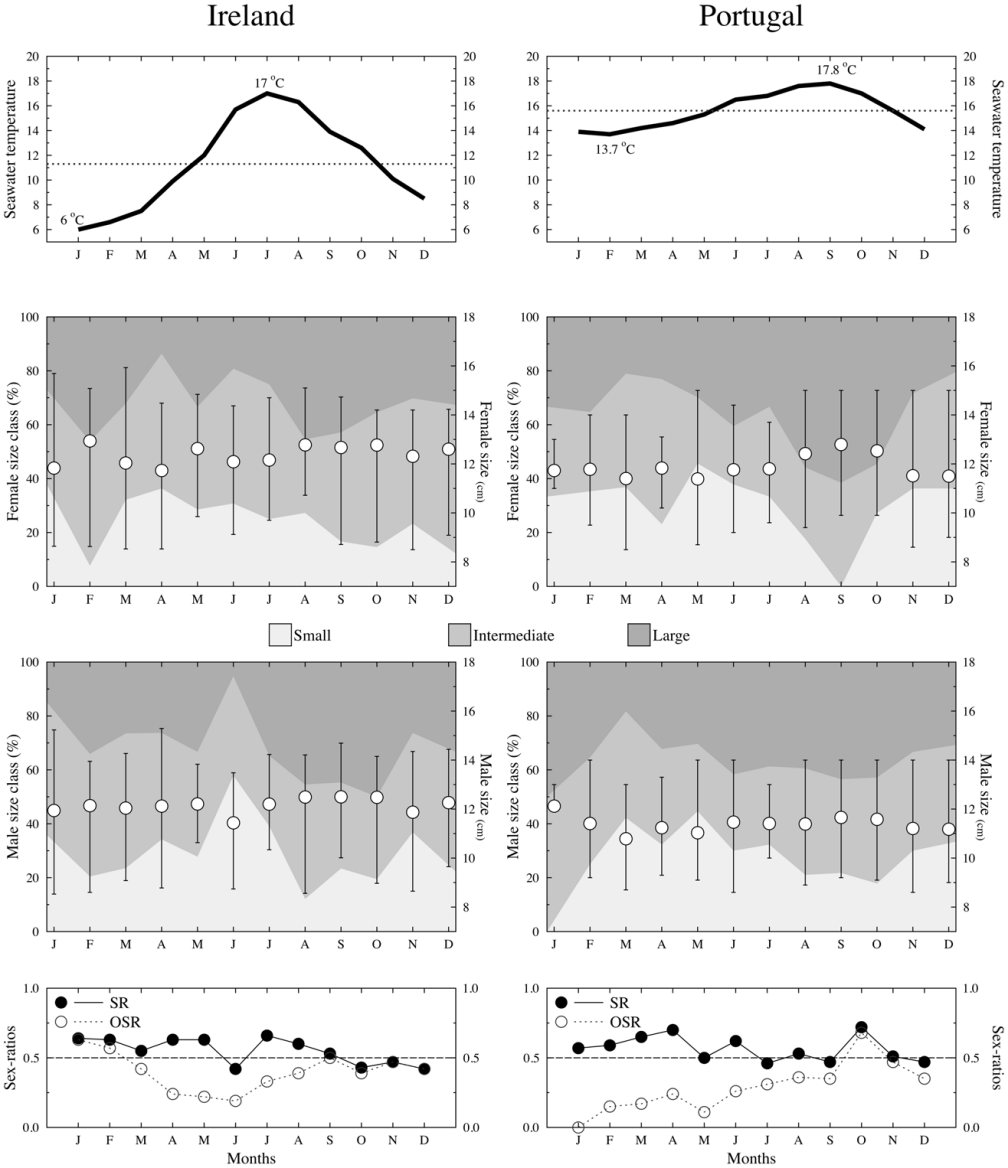
The surface seawater temperatures in the sampled areas is presented in the top plots, highlighting the average (dotted line) as well as the highest and lowest temperature values. The second and third plot rows depict the monthly average size of sexually mature female and male worm pipefish, respectively, with bars indicating the maximum and minimum values. The filled areas in the background show the monthly percentages of large, intermediate, and small individuals. The bottom plots represent the monthly variation in sex ratios (full circles depict the sex ratio while open circles show the operational sex ratio). Values for the Irish population are presented in the left column while those from the Portuguese population are shown in the right column.

As expected, seawater temperatures were much lower in the Irish sampling location, especially during the winter (Figure 3). In northern Portugal, the average monthly seawater temperatures did not drop below 13.7°C. Nevertheless, the maximum temperatures registered were similar, although peaking slightly later in the year in Portugal relative to Ireland (Figure 3). Once temperatures rose, smaller males and females were outnumbered by larger individuals at both sites (Figure 3).

The sex ratio (Figure 3) tended to be slightly male biased both in Ireland (average = 0.551, *SD* = 0.094, *N* = 12) and Portugal (average = 0.566, *SD* = 0.091, *N* = 12), although no significant differences were observed when monthly captures of males and females were contrasted within each location (Wilcoxon matched pairs test, Portugal: *N* = 12, *Z* = 1.608, *P* = 0.108; Ireland: *N* = 12, *Z* = 1.451, *P* = 0.147). Additionally, no differences were observed when comparing the calculated monthly sex ratios between the two locations (Sign test: *N* = 12, *Z* = −0.289, *P* = 0.773). The OSR differed significantly between the two locations (Sign test: *N* = 12, *Z* = 2,021, *P*<0.05), with a more female biased sex ratio in the Portuguese population, especially during the first half of the year (January to June; Figure 3).

**Figure 4:**
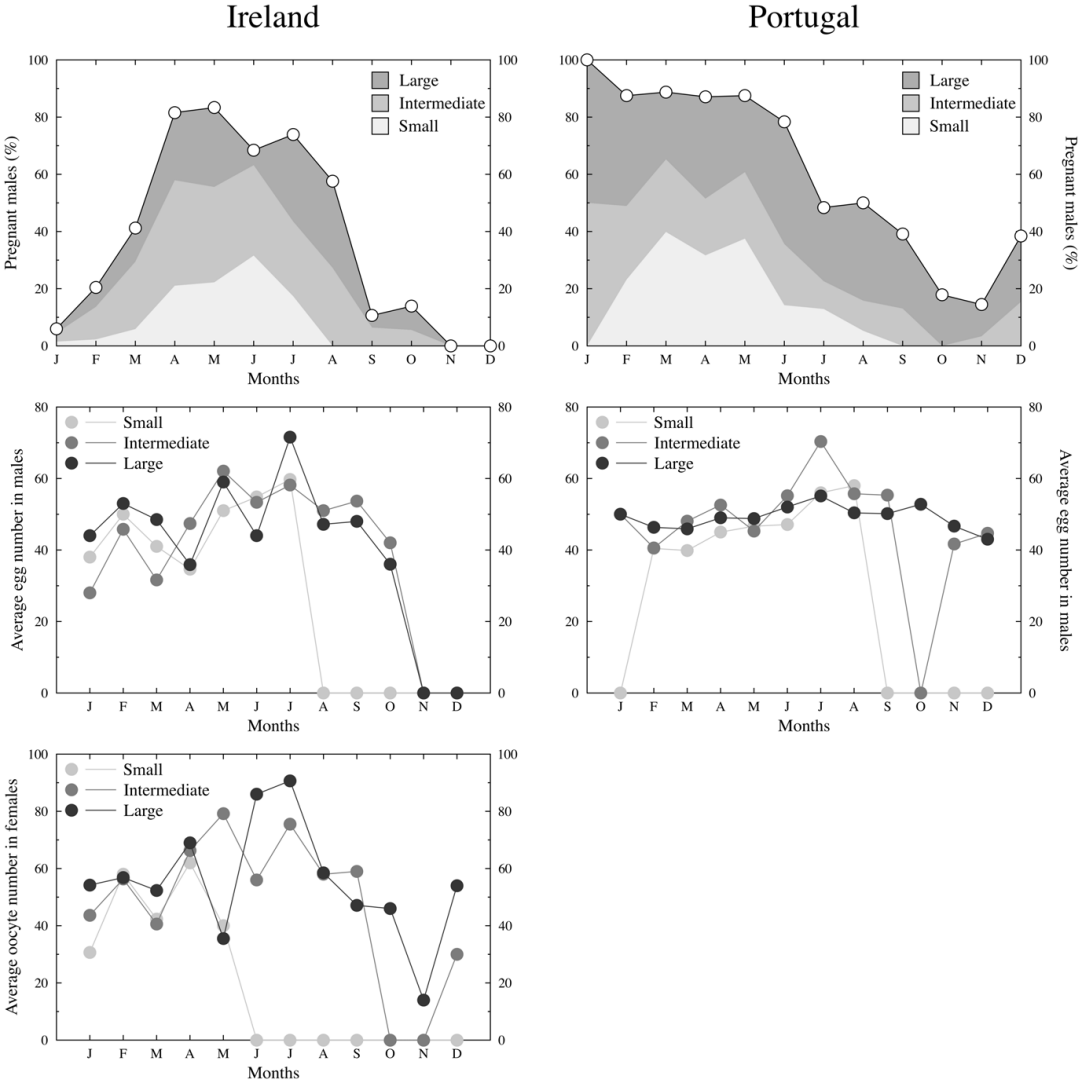
The monthly percentage of *Nerophis lumbriciformis* pregnant males in Ireland (left column) and Portugal (right column), where the filled areas depict the relative contribution of either large, intermediate or small individuals (top row). Middle row plots show the monthly average number of eggs carried in large, intermediate or small male's incubating surface. Bottom row plots illustrate the monthly average number of oocytes in large, intermediate and small Irish females.

Small males, although present in the intertidal (Figure 3), ceased to reproduce when water temperatures increased, as evidenced by the absence of small pregnant males in the intertidal (Figure 4). This pattern was similar at both sites, with smaller individuals interrupting reproduction for nearly 5 months (Ireland: August to December; Portugal: September to January). Larger males, on the other hand, continued to reproduce the entire year (with the exception of November and December in Ireland, when reproduction stopped altogether). During the seven months that large, intermediate, and small males reproduced, no difference was observed in the average egg number carried by pregnant males (Friedman ANOVA; Ireland (*N* = 7, *DF* = 2): *χ^2^* = 2.571, *P* = 0.276; Portugal (*N* = 7, *DF* = 2): *χ^2^* = *2*, *P* = 0.368; Figure 4). Also, the standardized selection differentials (S_males_) calculated for the period when all male classes were simultaneously breeding in both sampled populations (February to July) did not differ between sites (Wilcoxon matched pairs test, *N* = 6, *Z* = 1.782, *P* = 0.07). Interestingly, in Ireland (no data is available from Portugal) smaller females also stopped producing mature oocytes from June onwards (Figure 4). No differences in the average number of mature oocytes present in the ovaries were observed between large and small females within the 5 months (January to May) when both size classes were reproducing (Kruskall-Wallis ANOVA (2,87); *χ^2^* = 3.286, *P* = 0.63).

Pregnant males in Ireland carried on average 49.6 eggs (*SD* = 17.1, *N* = 145) while in Portugal the mean value was 47.5 (*SD* = 12.42, *N* = 307), a non-significant difference (Mann Whitney *U* = 20430, *P* = 0.159). There was, however, a significant difference in egg size (Mann Whitney *U* = 2070, *P*<0.001) with males from Portugal carrying larger eggs (average = 1.333 mm, *SD* = 0.126 mm, *N* = 84) than those in Ireland (average = 1.208 mm, *SD* = 0.139 mm, *N* = 109).

No significant relationship between male length and egg number was observed in the Irish population [Pearson product moment correlation coefficient (PPMCC), *N* = 142, *R* ^2^ = 0.0163, *P* = 0.129]. Similarly, a lack of trend is observed when analysing the relationship between male length and egg size (PPMCC, *N* = 109, *R* ^2^ = 0.005, *P* = 0.473). In Ireland, only males larger than 10 cm engaged in reproductive activity. In Portugal, however, the minimum size for a pregnant male was 8.5 cm. If only Portuguese males larger than 10 cm are included in the analysis (mimicking what happens in Ireland), size and egg number also did not correlate significantly (PPMCC, N = 243, *R* ^2^ = 0.0006, *P* = 0.711). Likewise, no relationship was observed between Portuguese male size (males >10 cm) and egg size (PPMCC, N = 55, *R* ^2^ = 0.030, *P* = 0.208). However, this result changed when Portuguese males smaller than 10 cm were included: a weak but significant positive correlation emerged between male size and egg number (PPMCC, N = 276, *R* ^2^ = 0.034, *P*<0.005), and also between male size and egg size (PPMCC, N = 63, *R* ^2^ = 0.078, *P*<0.05).

**Figure 5:**
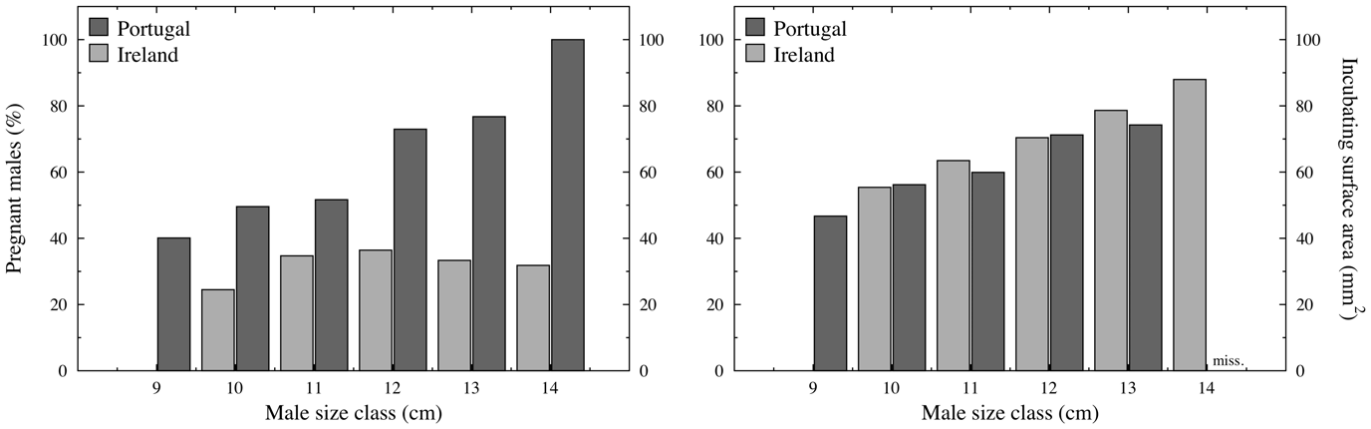
The annual percentage of pregnant males according to size class (left plot) and the average brooding surface area (right plot), in Ireland (light grey) and Portugal (dark grey; “miss.” indicates missing values).

In Ireland, there was no significant alteration in the percentage of pregnancy within each size class when considering the entire year (Figure 5, PPMCC, *N* = 5; *R^2^* = 0.508; *P* = 0.176). In Portugal, on the other hand, there was a strong correlation between male size class and pregnancy (PPMCC, *N* = 6; *R^2^* = 0.935; *P*<0.005), with the largest males always being pregnant. Thus, the slopes calculated for the Irish and Portuguese populations differed significantly [*F*(1,7) = 18.145; *P*<0.005].

When analysing the area of the incubating surface, fish in the same size class did not differ between locations (Wilcoxon matched-pairs test, *N* = 4; *Z* = 1.461; *P* = 0.144). A positive correlation between size and incubating surface was observed both in Ireland (PPMCC, *N* = 5; *R* ^2^ = 0.903, *P*<0.05) and Portugal (PPMCC, *N* = 5; *R* ^2^ = 0.971, *P*<0.005), with larger males presenting larger incubating surfaces. The calculated regressions for the two sampled locations did not differ in slope [*F*(1,6) = 0.680; *P* = 0.441] or in intercept [*F*(1,7) = 5.034; *P* = 0.06]. Nevertheless, there was a significant difference in the percentage of the occupied brooding surface (in pregnant males), when contrasting the sampled locations (Mann Whitney *U* = 1271, *P*<0.001, *N*_Portugal_ = 80, *N*_Ireland_ = 50), with males in Portugal presenting less free space (Ireland: average occupied brooding surface = 73.95%, *SD* = 20.11; Portugal: average occupied brooding surface = 85.97%, *SD* = 13.32).

Finally, when analyzing the length of male and female worm pipefish, both in Ireland and Portugal, significant differences were observed both between sexes [*F*(1,1731) = 32.578; *P*<0.001] and locations [*F*(1,1731) = 151.703; *P*<0.001]. A tendency for an interaction between sex and location [*F*(1,1731) = 4.976; *P* = 0.092] suggests that sexual size dimorphism was less prominent in the Irish population (SSD_Ireland_ = 0.29 mm; SSD_Portugal_ = 0.47 mm).

## Discussion

In previous works [22], [33], *N. lumbriciformis* breeding season has been delineated by the overall percentage of pregnant males caught in the intertidal in different months. Observations of a Portuguese population indicated a peak of reproductive activity in male pipefish correlated with lower water temperatures (December to June [22], [33]). It is now apparent that previous findings overlooked a considerable substructure in the reproductive behaviour of this pipefish. Large and small individuals clearly differ on the timing and length of the breeding season. This substructure is not peculiar to the Portuguese population, as it also was observed in the Irish population of the worm pipefish showing that size is indeed a central variable worth taking into account when addressing pipefish reproduction.

### The effect of body size in worm pipefish reproduction

The intertidal area acts primarily as a mating arena [10], but sexes as well as size classes use it differently over time. For instance, smaller individuals had a truncated breeding season in comparison to larger pipefish, which reproduced year-round. This pattern was observed in both sampled populations, only differing in that reproduction in Ireland stopped entirely during two months at the end of the year. Smaller males reproduced mainly during the first half of the year, up to the point where seawater temperature reached its maximum value. Shortly before, smaller females also stopped producing oocytes, at least in Ireland. From then onwards, larger fish predominated in the intertidal and continued to reproduce, presumably among themselves. Since smaller individuals were still observed in the intertidal, albeit not reproducing, perhaps some physiological barrier imposed by high temperatures could only be overcome by larger individuals, granting them a competitive advantage that translates into: i) an extended breeding season, ii) faster development of embryos (incidentally allowing for an increased number of pregnancies during the breeding season; see [21]) and iii) a possible increased survival of the offspring [at least in the south: [33] showed that recruitment peaks occur shortly after the warmest months, suggesting that pipefish born during the summer have higher probability of recruiting to coastal areas].

*N. lumbriciformis* is strongly sex-role reversed, with the larger, more ornamented females actively competing for access to males, which are the choosier sex [19]. Given the observed male choosiness and probable female fitness gains from reproducing with larger males (larger males have for instance larger incubating surfaces which ensures enough space for an egg batch), we would expect that high quality males would have preferential access to the larger, high quality females, and would thus bear more and larger eggs, i.e., we expected size assortative mating. Indeed, in *Syngnathus abaster* [34] or *S. typhle* [35], larger females tend to deposit more and larger eggs preferentially on high quality males. In contrast, in *N. lumbriciformis* when comparing the number and size of eggs carried by large or small males, no obvious differences were found in either population. Three possibilities open up: i) if egg size and/or number do vary with female size, there is no size assortative mating, or ii) egg size and/or number is independent of female size or iii) egg size and/or number is dependent on female size but only up to a specific size class (10 cm, in the case of the worm pipefish), rendering size assortative mating cryptic in larger fish.

### The minimum size threshold

The third hypothesis regarding the minimum size threshold is much more probable if we take into account a feature that occurs only in the Portuguese population: males smaller than 10 cm reproduce. Yet, these very small males carry fewer and smaller eggs, and when adding the small male data to the otherwise non-significant correlations from large males, a positive correlation between male size and egg number/size emerged. Although the obtained correlation coefficient was so small that it could be argued not to be biologically relevant, the addition of a few small individuals still changed the slope from zero, so the small males made a substantial difference. This implies that there is indeed a threshold (around 10 cm) above which size assortative mating ceases or becomes unnoticeable through egg size analysis. Actually, the smaller males have brooding surfaces that have insufficient capacity to accommodate the average number of eggs normally incubated by male worm pipefish. In *N. lumbriciformis*, females produce a large batch of eggs that are passed in one go onto the males' brooding surface [19]. If the egg batch surpasses the male incubating capacity, surplus eggs invariably drop to the bottom and are ultimately lost. Thus, females should avoid small males with very small brooding areas.

The “minimum size threshold” hypothesis may also explain why there are no very small pregnant males in Ireland. Males might be postponing reproduction by maximizing body size prior to first reproduction, a strategy that has already been suggested for other pipefish species [36], especially in areas where breeding seasons are shorter and depress opportunities for reproduction even with potential “leftovers”. Females in the Irish population seem to follow the exact same “minimum size threshold”, as only one female smaller than 10 cm was ever found containing mature oocytes.

### Size and latitude

Recently, Wilson [37] noted that Bergmann's rule acts differentially in syngnathids. Body size seems to be uncorrelated with latitude in the monogamous seahorses while a variation consistent with Bergmann's rule was observed in the polygynous pipefish *Syngnathus leptorhynchus*. Wilson [37] interpreted these findings (increase in body size with latitude) as a mechanism capable of maintaining the potential reproductive rates of males despite lower seawater temperatures (more eggs offsetting the extended brooding periods). Accordingly, a link between fecundity selection and Bergmann's rule was hypothesized. Bergmann's rule may also apply to *N. lumbriciformis*. Individuals from Ireland were larger than those captured in Portugal, where seawater temperatures are generally higher. However, the underlying reason may differ from that proposed for *S. leptorhynchus*. Just like in *N. ophidion* [38], a *N. lumbriciformis* male accepts eggs from only one female during a breeding episode [10]. Thus, being unable to collect eggs from multiple females, an increased male size (i.e., a larger brooding space) would only offset a prolonged pregnancy if females from the north were able to deposit more eggs than their southern counterparts. However, this was not the case as no difference in the egg number carried by pregnant males was observed between the two populations. The fact that pipefish in the Irish population attained larger sizes may instead be linked to latitude-specific energetic adaptations [39]. Alternatively, given that the smaller pipefish experience a shorter breeding season, attaining larger sizes might potentially increase individual fitness not during the extent of a pregnancy, but along the full extent of the breeding season.

Even though worm pipefish are smaller in Portugal, the size difference between males and females, or sexual size dimorphism (SSD), is 61% larger than in the Irish population, suggesting that Rensch's rule, at the intraspecific level, seems to apply in this pipefish (size dimorphism decreases with increasing average body size, when the female is the larger sex; see [13], [14]). So, what may be the underlying causes explaining this difference in SSD? Although adult sex ratios were slightly male biased in both populations, a highly female biased operational sex ratio emerged, consistent with the evident sex role reversal. Moreover, the skew towards females was stronger in the Portuguese population, possibly increasing female-female competition and intensifying the degree of sex role reversal, all concordant with the higher SSD observed at this site. Although we were not able to directly measure the pressure of sexual selection on females (we were not equipped to quantify female reproductive success as males carry eggs, which cannot be assigned to a specific female without using molecular techniques for parentage analysis), the similarity in the calculated male selection differentials suggests that the registered differences between the two sampled populations should derive from a stronger selective pressure on females, as expected for a sex role reversed species. Thus, if southern females do experience a stronger sexual selection pressure, we predict a larger reproductive investment from them (e.g. larger eggs). This challenges Rass's rule (i.e., an increase in egg size with a decrease in water temperature, see [15]), but is exactly what we found: females in Portugal seem to invest more in reproduction not only by mating more often (some females return to the intertidal within one breeding season; see [10]) but also by laying larger eggs when compared to those from Ireland. These larger eggs also translated into fuller male breeding surfaces in the Portuguese population.

This study depicts the dynamic nature of reproduction in natural populations, across latitudes, simultaneously along the breeding season and as fish mature and continue to grow. Even though the Portuguese and the Irish populations inhabit locations with very different water temperature regimes, they exhibit considerable similarities in reproductive parameters: occupancy of the breeding grounds, densities, extent of the breeding season and size-related reproductive substructure were all remarkably alike. The most obvious of these similarities is the existence of a well-defined substructure in reproductive activity, apparently similar on both sexes. This size-related structure, where larger individuals reproduce for longer periods, seems to be dependent on a high temperature threshold, rather than on the low temperatures that vary heavily according to latitude. High seawater temperatures seem to mark a clear reproductive boundary for small individuals, which stop to breed entirely. Thus, at least theoretically, increasing seawater temperatures will reduce the number of breeding individuals, by delaying the first reproduction of recently matured fish. These findings advocate that special attention should be devoted to the description of a species reproductive ecology as the existence of size-related substructure can be easily overlooked when analysing pooled data from an entire population. If, as predicted in the coming years, seawater temperature continues to rise due to climate change, the size-dependent reproductive substructure observed within worm pipefish populations might be seriously affected, with (yet) unpredictable consequences.

Even though the major differences between the two geographically distant populations might stem from either genetic or environmental factors (e.g. predation pressures, light or feeding regimes), the observed disparities on size at first reproduction, female egg investment or degree of SSD, can be easily correlated to an interplay between water temperature and OSR. While intrinsically simple and thus less capable of providing very precise estimations of the actual intensity of sexual selection, the OSR substantiated the observed differences within and between the sampled populations, suggesting a stronger pressure of sexual selection in the south. As the extent of the breeding season as well as fecundity are usually reduced in cold waters [11], we were expecting to find support for an enhanced opportunity for sexual selection in the north. Nevertheless, we found evidence for the exact opposite. Since available males limit females, not because there is little time to mate but because enough vacant brooding surfaces are scarce (especially when temperatures rise and small males cease to reproduce), sexual selection pressure is sure to act strongly on females. In fact, if the limited sex has the ability to reproduce, either continuously or recurrently, during the entire breeding season (similarly to what is observed for the closely related species *N. ophidion*, [40]), an increased opportunity for sexual selection might arise from the continuous need to compete for available partners on protracted breeding seasons, under strongly biased OSRs.

Even though many studies have addressed the consequences of climate change on a number of ecological processes [41], there is still limited evidence of the potential impact of climatic variation on sexual selection. Some of the examples recently put forward, either on mammals [8] or birds [42] with conventional sex roles, point to the gradual dilution of the pressure of sexual selection, resulting from new mating opportunities for males that previously had lesser chances of reproducing. In our study, involving a sex-role reversed species, it seems that higher temperatures might not necessarily translate into weaker sexual selection.

Predicting how climatic variation will ultimately influence the strength of sexual selection is undeniably a complex issue, but individually looking at a species' mating system might be a good starting point. For instance, polygynic mating systems, where the intensity of sexual selection on males is high [43], may be greatly impacted by climatic variation if dominant males are no longer able to secure female monopolization. In this case, the observed mating system will slide towards polygamy, where the intensity of sexual selection is expectably weaker. Conversely, on the other side of the spectrum, a polyandric mating system will also move towards polygamy if females are no longer able to monopolize males. In short, if climate change depresses the opportunity to monopolize mating opportunities, a depression in the intensity of sexual selection can be expected, irrespectively of sex roles. Alternatively, if climatic variation selectively depresses the fitness of those individuals with characteristically worst reproductive performance, then sexual selection intensity is expected to increase. Additional research on the geographical variation of the reproductive ecology of the worm pipefish, as well as other species, will surely help describe, with even greater detail, the spatial and temporal dynamics of sexual selection pressure.
